# Using the microbiome in clinical practice

**DOI:** 10.1111/1751-7915.13971

**Published:** 2021-11-12

**Authors:** Sondra Turjeman, Omry Koren

**Affiliations:** ^1^ Azrieli Faculty of Medicine Bar‐Ilan University Safed Israel

## The microbiome and host health

Over the past 20 years, researchers have uncovered endless ways in which microbiota are associated with host health and behaviour. The microbiota throughout the body – gut, oral cavity, nasal cavity, skin, vagina – all function in a delicate balance aiding in digestion, pathogen deterrence and general host homeostasis. Microbial dysbiosis (a shift away from a balanced composition) is also implicated in a number of conditions and diseases ranging from obesity (Turnbaugh and Gordon, 2009; Ley, 2010) to inflammatory bowel diseases (Spor *et al*., 2011; van der Giessen *et al*., 2020) to autoimmune diseases (Shamriz *et al*., 2016; Neuman and Koren, 2017) to behavioural and mental health disorders, such as stress (Werbner *et al*., 2019) and Alzheimer’s disease (Cryan *et al*., 2020). There is constant bi‐directional communication between the microbiota and the host, and the microbiota both drives health complications in the host and responds to them. Thus, the microbiome already does and will continue to play a role in health and healthcare best practices. In this perspective, we highlight the anticipated role of the microbiome and microbiome‐driven applications in modern medicine.

## Diagnosis

To date, the microbiome, or more accurately, microbial dysbiosis, has been implicated in a range of human diseases and conditions (Lynch and Pedersen, 2016), and researchers agree that bacterial activity associated with metabolite production and breakdown can drive disease pathologies (Vernocchi *et al*., 2016; Van Treuren and Dodd, 2020). Researchers are now actively striving to determine how specific bacterial taxa and metabolite profiles relate to metabolic pathway regulation and human health (Hidalgo‐Cantabrana *et al*., 2017; Tett *et al*., 2021). To that end, specific bacterial taxa or metabolites are being pinpointed in disease pathogenesis. The natural extension of taxa‐ or metabolite‐focused studies is to identify relevant biomarkers for disease diagnosis. Specifically, unique microbial signatures have been found in blood and tissues of cancer patients, allowing identification of not only sick from healthy individuals but also of cancer type among sick individuals (Poore *et al*., 2020). Additionally, a panel of 30 microbial biomarkers was identified for diagnosing hepatocellular carcinoma based on faecal microbial composition collected from hepatocellular carcinoma patients (Ren *et al*., 2019). Salivary microbiome‐derived biomarkers can also be used diagnostically (Kaczor‐Urbanowicz *et al*., 2017). Two bacterial species identified in the oral microbiome of pancreatic cancer patients, *Neisseria elongata* and *Streptococcus mitis*, can differentiate these individuals from healthy, age‐matched controls with extremely high accuracy (Farrell *et al*., 2012). Metabolite profiles are also relevant diagnostic tools with applications already seen for cancers (Beger, 2013) and Alzheimer’s disease (Paraskevaidi *et al*., 2020). Other diagnostic targets include identification of quorum sensing/biosensing by‐products (Miller and Gilmore, 2020) which could reveal biofilm presence and bacterial virulence or specific pathologies (De Spiegeleer *et al*., 2020).

The use of non‐specific microbiota screening by general practitioners and family physicians could serve as a healthy‐visit tool to identify hidden pathologies. Or a microbiome panel could be offered, similar to how genetic testing (SNP testing for specific mutations) is used to identify individuals’ risks for genetic disorders or couples’ risks of passing mutations to their offspring. General microbial or metabolic testing could be a relatively inexpensive way to screen for diseases that once required specialist visits and invasive or expensive procedures. Mail‐in testing could also greatly increase healthcare access in less‐serviced areas, not only improving individual well‐being but also reducing strain on an already fragile healthcare system. While specific panels for a range of diseases have already been identified, in the next 15 years, we see general screening gaining momentum in non‐specialty medical settings.

## Prediction

Researchers have already demonstrated the applicability of using microbiota composition and metabolite profiles to diagnose diseases, but impressively, they have also demonstrated its utility in predicting future disease onset, even before diagnosis using current medical best practices, as demonstrated for gestational diabetes (Pinto *et al*., 2021), coronary artery disease (Zheng *et al*., 2020) and celiac disease (Leonard *et al*., 2021). Machine learning (ML) tools are now being adapted in a range of studies to predict disease onset based on pre‐dysbiosis, microbial imbalances that may serve as precursors driving specific diseases, and other patient characteristics. Thus, as with the described diagnostic measures above, we expect predictive models with microbial or metabolite profile inputs to serve as relatively non‐invasive clinical tools in the coming decade.

Machine learning models can include microbial features (specific taxa) and their abundance as well as additional biomarkers – metabolites, cytokines, hormones – genes and other risk factors (historical, environmental and behavioural) to predict disease onset (Marcos‐Zambrano *et al*., 2021). The addition of microbial data has shown increased efficacy compared to typical demographic‐based predictors, and with exploding research in the field of host–microbiome interactions, the range of diseases and disorders that can be predicted will continue to grow. Thus, modelling efforts could be combined with the above‐described microbial screening panels to not only diagnose patients but also predict future maladies, providing opportunities for early intervention.

Even more impressive, new research suggests that microbiota data can also be applied to predict host responsiveness to specific treatments, including those for cancers (Veziant *et al*., 2021; Yi *et al*., 2021), *Clostridioides difficile* infection (Khanna *et al*., 2016), rheumatoid arthritis (Artacho *et al*., 2021), bariatric surgery (Ben Izhak *et al*., 2021) and inflammatory bowel diseases (Shaw *et al*., 2016; Ananthakrishnan *et al*., 2017). For example, in the case of rectal cancer, patient response to neoadjuvant chemoradiotherapy (nCRT) was predicted with > 73% accuracy in a validation cohort using 10 microbial taxa differentially expressed between responders and non‐responders as predictors (Yi *et al*., 2021). Additionally, a study of responders and non‐responders with melanoma receiving anti‐programmed cell death 1 protein (PD‐1) immunotherapy revealed both differentially abundant bacterial taxa and different functionality profiles between the groups based on metagenomics and pathway analysis (Gopalakrishnan *et al*., 2018). The microbiome is implicated in a range of diseases and host states, so it is only expected that it would be implicated in treatments as well. Accordingly, current research points to the utility of microbiota screening when planning treatment for a range of diseases, and a standardized ML model that accounts for patient characteristics, disease expression and microbiota composition could change the way we approach disease treatment. If patient responsiveness to treatments can be predicted from non‐invasive faecal or saliva sampling, healthcare can be tailored to patient needs, both improving efficacy, and decreasing burdens – financial, mental and otherwise – associated with extended treatment plans and low success rates.

## Treatment

We have described how microbiota and metabolite profiles can be used to diagnose diseases, predict future onset and even predict treatment success rates. It is only logical, then, that microbiota manipulation or supplementation can aid in treatment of a range of diseases. The study of how drugs and host microbiota interact has come to be known as pharmacomicrobiomics. Pharmacomicrobiomics typically focuses on drug metabolism and treatment efficacy as they relate directly to the microbiota and indirectly to the metabolic processes driven by the microbiota. Early evidence in cancer models suggests that response to treatment can be improved through specific microbiota manipulation. In one study of melanoma in a murine model, administration of several *Bifidobacterium* species increased anti‐programmed cell death ligand‐1 therapy efficacy, and even when administered alone without therapy, these bacteria increased tumour control (Sivan *et al*., 2015). A second study of melanoma in a mouse model found beneficial effects of *Bacteroides fragilis* on host responsiveness to and efficacy of CTLA‐4 blockade, and thus increased antitumor effects (Vetizou *et al*., 2015). Both studies are based on preliminary findings of differences in the microbiomes of responders and non‐responders and show how successful application of targeted treatment – here inoculation with specific bacterial strains – enhances treatment efficacy.

Once differences in responders and non‐responders can be traced to the microbiota, treatments can be developed and applied in a stratified manner (Zhang *et al*., 2019). Pre‐screening patients’ microbiota to determine if they are good candidates for a certain treatment may become standard practice; it is no longer technologically complex, and 16S rRNA gene sequence surveys are becoming less financially limiting, though rapid screens are still lacking. As discussed above, bacterial inoculations can be highly customized, introducing a single bacterial strain or species to patients prior to or concurrent with standard treatments, but identifying specific bacterial taxa suited to each of the many different therapeutics used to treat the wide range of complex human diseases remains a challenge. There is evidence, however, that total faecal microbiota transplant (FMT) from responders, rather than specific bacterial inoculation, can also improve treatment outcomes. As recently demonstrated in a murine model, FMT from donor mice with microbiota that demonstrated antitumor effects to mice of the *same strain* but sourced from a different animal facility and hosting a different microbiota reduced tumour growth to a similar degree as antibodies targeting PD‐L1 (Sivan *et al*., 2015). Studies in humanized mice – those that received FMTs from human responders and non‐responders – also show beneficial effects on treatment success following FMT from responders (Sivan *et al*., 2015; Gopalakrishnan *et al*., 2018; Matson *et al*., 2018; Routy *et al*., 2018). A range of studies in humans are underway to determine the effects of FMT on patients who are non‐responders to cancer treatments (McQuade *et al*., 2019), and preliminary results from two pilot studies in humans revealed that FMT can increase responsiveness to anti‐PD‐1 immunotherapy (Baruch *et al*., 2021; Davar *et al*., 2021). 

 Beyond improving treatment outcomes, microbiota‐based medicine could reduce unwanted effects of certain medicines. One study found that metastatic melanoma patients treated with ipilimumab who go on to develop colitis could be predicted by surveying their pre‐treatment microbiota (Chaput *et al*., 2017), and we have recently shown that the microbiome of patients who will gain weight following chemotherapy was different from that of those who will not (Uzan‐Yulzari *et al*., 2020). Additionally, studies have shown that antipsychotic use in children and adolescents, even when effective as therapeutics, can result in microbiota dysbiosis and associated microbiome‐derived weight gain (Bretler *et al*., 2019; Libowitz and Nurmi, 2021). Over the coming years, researchers should be able to exploit pharmacomicrobiomics not only to increase treatment efficacy but also to reduce unwanted side effects of therapeutics, making treatments with potentially debilitating side effects in a subset of the population more palatable and raising the appeal of treatments like antipsychotics, and even birth control, to individuals who are extremely hesitant because of associated weight gain.

## FMT Banks

As described above, microbiota manipulation, either via FMT or specific bacterial targets, can be used to treat or improve treatment of a range of diseases. Evidence of FMT to improve digestive function and reduce diarrhoea dates back thousands of years to 4th century China (Zhang *et al*., 2012) and has historically been practiced by Bedouins in Africa as well (Hanssen *et al*., 2021). More contemporary research, some of which is described above, shows the utility of FMT in modern medicine, and FMT as a treatment for recurring *Clostridioides difficile* is now commonly accepted following randomized clinical trials (van Nood *et al*., 2013). The question of the optimal FMT donor is still hotly contested, though. Faeces are, by nature, non‐sterile, and transfer of faecal matter from one individual to the next can have unwanted side‐effects if not screened properly. Thus, the option of autologous FMT (aFMT) is being explored (Hanssen *et al*., 2021). In this scenario, stool from the patient which was collected prior to disease onset can be used for FMT to reinitiate a healthy gut microbiota. Support for application of aFMT is seen in a recent study demonstrating that aFMT can help obese participants maintain weight loss following weight‐control diets (Rinott *et al*., 2021; Rinott *et al*., 2021). aFMT to treat disease, though, would require prior banking of faeces, something not common, at least in the year 2021. In the future, periodic faecal banking for future aFMT may become standard, or at least more common, just as cord blood banking has started gaining traction. Alternatively, donor banking may be more feasible for widespread application. To that end, a number of recommendations have been developed towards ensuring safe FMT, including patient and sample screening, labelling methods, and storage conditions (Saha and Khanna, 2021). Once banked, samples could theoretically be characterized and classified to highlight which would most likely improve certain disease states, improve treatment responsiveness and help with weight loss and ageing, even taking into account recipient characteristics (Schmidt *et al*., 2021). Down the line, we could even envision a world in which FMT from “star” donors would be used as probiotics are today to improve general well‐being and gut functionality, though as a one time or annual supplement rather than a daily one.

## Bacterial cocktails and personalized probiotics

Faecal microbiota transplant is a blanket approach for altering the microbiota, but probiotics (or in germ‐free models, mono‐colonization), prebiotics and postbiotics can offer better targeting and may reduce risks of infection that, though rare, have been associated with FMT (DeFilipp *et al*., 2019; Eshel *et al*., 2021). Once functionally relevant taxa and metabolites are identified from the sets of differentially abundant features that distinguish patients from healthy controls, bacterial or metabolite cocktails can be formulated in a disease‐specific manner with the goal of regaining metabolic homeostasis. Further, patient profile – specific microbial or metabolite deficiencies both related to and independent of disease state – can be incorporated into models to tweak or supplement disease‐specific cocktails. Such personalized approaches, that consider both disease state and specific patient characteristics, should not be too far off. As soon as models for disease prediction and treatment responsiveness are in use, taking modelling one step further towards personalized probiotic formulations is no longer a stretch.

Beyond direct treatment consequences, personalized microbiota‐based medicine could also be used to improve quality of life or lifespan. Custom probiotics could abound – to reduce weight, boost immunity during cold season, sharpen memory, improve fertility, aid in initial gut colonization and more. The microbiome is implicated in a range of developmental functions (Leclercq *et al*., 2017; DeFilipp *et al*., 2019; Champagne‐Jorgensen *et al*., 2020; Kayyal *et al*., 2020), diseases, mental disorders (Forsythe *et al*., 2016) and host states (Koren *et al*., 2012; Goldberg *et al*., 2020), some of which are mentioned above. Even ageing is associated with microbial dysbiosis (Binyamin *et al*., 2020), and FMT from young donor mice can counteract some of the ageing phenotype (Boehme *et al*., 2021). There is also early evidence that centenarians have significantly different gut microbiota than elderly and young individuals with relevant differences in metabolic function related to bile acid production (Sato *et al*., 2021). Another study in centenarians found that there are marked microbial shifts that occur in the seven‐month window prior to their passing (Luan *et al*., 2020). Thus, we might be able to increase longevity through targeted microbiota supplementation. Currently, probiotic formulas typically consist of lactic‐acid‐producing bacteria, such as lactobacilli and bifidobacteria, which are easily isolated from dairy products (Cunningham *et al*., 2021), but as knowledge of microbiota functionality expands, bacterial probiotic targets are also expanding to include *Roseburia intestinalis*, *Faecalibacterium prausnitzii*, *Eubacterium* spp.*, Bacteroides* spp. and *Akkermansia muciniphila,* and other taxa thought to confer benefits to human health (Brodmann *et al*., 2017; O'Toole *et al*., 2017; Cunningham *et al*., 2021). Following refinement of new probiotic therapeutics, termed next‐generation probiotics, clinicians and nutritionists could prescribe specific formulas for patients with known microbial deficiencies or who wish to improve certain gut or even overall host functionality.

## Conclusions

With the advent of next‐generation sequencing, untargeted metabolomics, transcriptomics and proteomics, specific functions can now more accurately be assigned to bacterial taxa, helping researchers to identify relevant target species for microbiota‐based therapeutics. And bioinformatics pipelines and machine learning algorithms are constantly being refined to better integrate this wealth of omics data towards clinical application. The field of microbiota‐based diagnostics and personalized or condition‐based microbiome‐related therapeutics will likely rapidly develop. With the advancement of these technologies, we will probably also see an increase in non‐essential therapeutics, including boutique and custom‐mixed probiotics specifically suited to the recipients’ endogenous microbiota and formulated to work on one or more quality‐of‐life targets. Overall, we can expect healthcare to benefit greatly from microbiome‐enhanced clinical practices, both in the rarer cases of non‐responders and for more widespread application in the general population in terms of screening towards early detection and intervention for a range of diseases. As the global population continues to age, researchers are constantly searching for new drugs and treatments; therapeutics, especially relatively inexpensive ones, associated with low risks (like faeces) will likely proliferate as independent treatments, as complementary treatments, and as preventatives.

## Conflict of interest

OK is a consultant to Mybiotics.
